# Anti-staphylococcal activities of lysostaphin and LytM catalytic domain

**DOI:** 10.1186/1471-2180-12-97

**Published:** 2012-06-06

**Authors:** Izabela Sabala, Ing-Marie Jonsson, Andrej Tarkowski, Matthias Bochtler

**Affiliations:** 1 International Institute of Molecular and Cell Biology, Trojdena 4, 02-109, Warsaw, Poland; 2 Max-Planck Institute of Molecular Cell Biology and Genetics, Pfotenhauerstr. 108, 01309, Dresden, Germany; 3 Department of Rheumatology and Inflammation Research, University of Gothenburg, Guldhedsgatan 10A, S-413 46, Gothenburg, Sweden; 4 Institute of Biochemistry and Biophysics, Polish Academy of Sciences, Pawinskiego 5a, 02-106, Warszawa, Poland

## Abstract

**Background:**

Lysostaphin and the catalytic domain of LytM cleave pentaglycine crossbridges of *Staphylococcus aureus* peptidoglycan. The bacteriocin lysostaphin is secreted by *Staphylococcus simulans* biovar *staphylolyticus* and directed against the cell walls of competing *S. aureus*. LytM is produced by *S. aureus* as a latent autolysin and can be activated *in vitro* by the removal of an N-terminal domain and occluding region.

**Results:**

We compared the efficacies of the lysostaphin and LytM catalytic domains using a newly developed model of chronic *S. aureus* infected eczema. Lysostaphin was effective, like in other models. In contrast, LytM was not significantly better than control. The different treatment outcomes could be correlated with *in vitro* properties of the proteins, including proteolytic stability, affinity to cell wall components other than peptidoglycan, and sensitivity to the ionic milieu.

**Conclusions:**

Although lysostaphin and LytM cleave the same peptide bond in the peptidoglycan, the two enzymes have very different environmental requirements what is reflected in their contrasting performance in mouse eczema model.

## Background

The problem of growing antibiotic resistance has been solved only in part by the introduction or reintroduction of new antibiotics (such as the quinupristin/dalfopristin Synercid [1] and the oxazolidinones [2]). Peptidoglycan hydrolases represent an alternative to small molecule antibacterials, despite concerns relating to immunogenicity, the release of proinflammatory components during bacteriolysis and the development of resistance [3]. The peptidoglycan endopeptidases lysostaphin and LytM cleave the characteristic pentaglycine crossbridges of *S. aureus* peptidoglycan [4-6] and are therefore of interest as potential antistaphylococcal agents.

**Figure 1:**
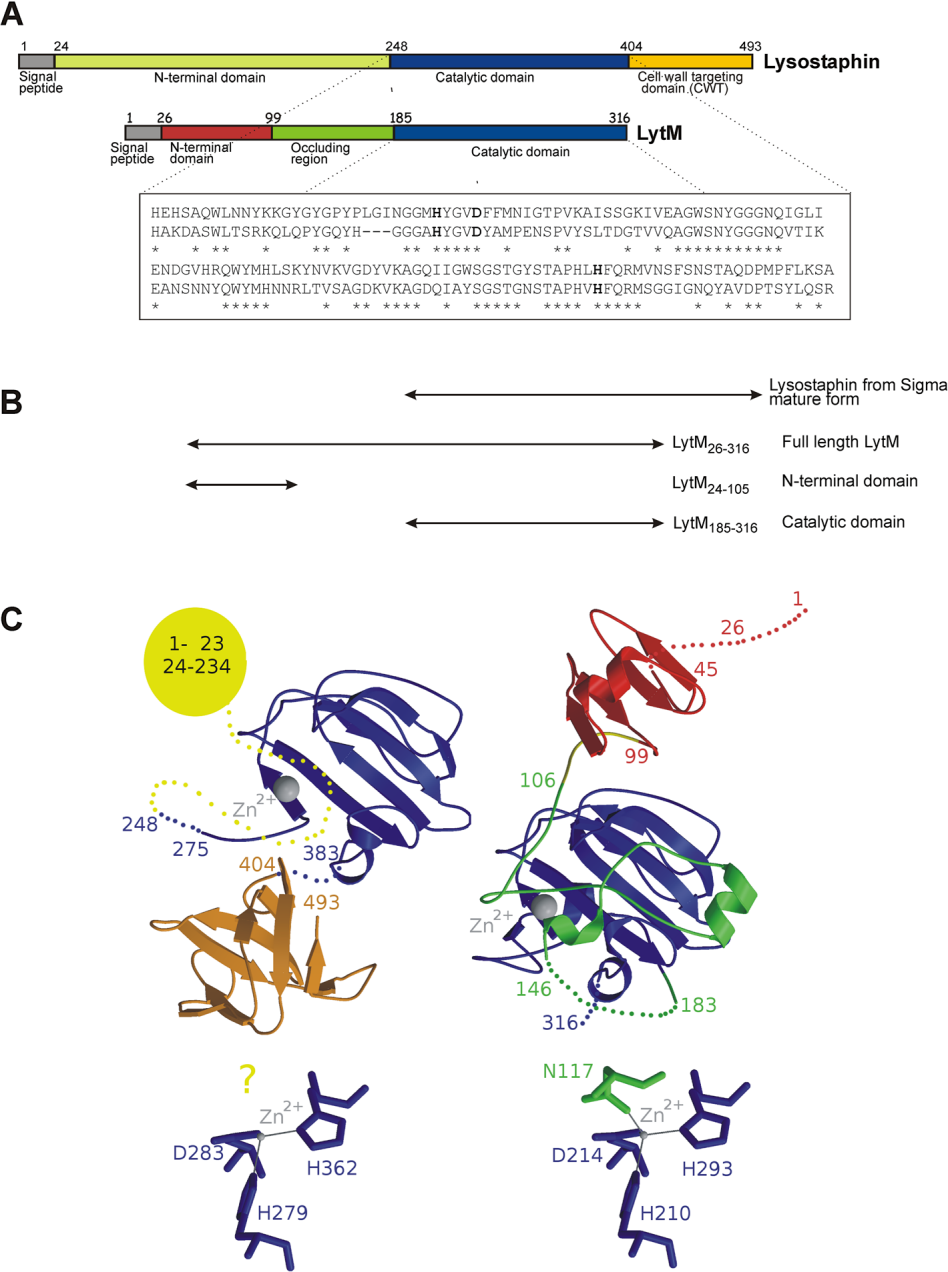
**Domain organization of preprolysostaphin and full-length LytM. (A)** Schematic representation of the domain organization of preprolysostaphin and full-length LytM. The alignment shows the high similarity of the two proteins in the region of the catalytic domain. The Zn^2+^ ligands of the mature forms in the Hx_3_D and HxH motifs are highlighted in bold. Those in the Hx_3_D motif are separately changed to alanines in the mutationally inactivated LytM variants. **(B)** Schematic representation of lysostaphin, LytM, and the LytM fragments that are used for this study. **(C)** Overall (top) and active site region (bottom) representations of the three-dimensional structures of preprolysostaphin (left) and full-length LytM (right). The left overall model was generated by the SWISSPROT server based on PDB entries 1QWY [12] and 1R77 [13]. The relative orientation of the catalytic and CWT domains is unknown and was chosen arbitrarily. It is not known whether the proregion repeats assume a defined structure or remain unstructured. The right overall model is an experimental structure directly based on PDB entry 1QWY [12].

Lysostaphin (Figure 1) is produced by *Staphylococcus simulans* biovar *staphylolyticus.* The secreted preproprotein is synthesized with a leader sequence, proregion, catalytic domain, and the cell wall targeting domain (CWT) [7]. The low complexity proregion consists of a variable number of stereotypical repeats (sequence [8]. It can be cleaved off *in vivo* by extracellular cysteine protease [9] to release the mature form, which is often simply called lysostaphin and is commercially available. Mature lysostaphin consists of the catalytic and CWT domains. The catalytic domain belongs to MEROPS family 23 in clan MO [10] and can be classified with the LAS metallopeptidases [11]. Sequence alignments suggest that the single Zn^2+^ ion in the active site is coordinated by His279, Asp283 and His362 (numbering according to Swiss-Prot entry P10547) and a water molecule. As the name implies, the CWT domain anchors the protein to cell walls [9] (Figure 1).

The biological role of lysostaphin is well established. The (mature) protein is inactive against the producer organism, but very effective in cleaving *S. aureus* cell walls [14]. This property has made the enzyme attractive as an antibacterial agent [15-21]. The protein has been applied to disrupt *S. aureus* and *S. epidermidis* biofilms on artificial surfaces [22] and has also been tested as a coating for catheters [23]. In a mouse model, lysostaphin has been used to eradicate *S. aureus* biofilms from a catheterized jugular vein [24] and also for treatment of systemic infections [25]. In a cotton rat model, a lysostaphin cream has proven effective in eradicating *S. aureus* nasal colonization [26]. In humans, lysostaphin has been used on an experimental basis to treat methicillin-resistant *S. aureus* aortic valve endocarditis [27]. As the elimination of *S. aureus* carriage in hospital staff is demonstrably effective in reducing infection rates in surgical patients and those on hemodialysis [28], a lysostaphin cream to treat infected, but asymptomatic hospital staff, has potential.

*Staphylococcus aureus* LytM (Figure 1) is an autolysin under the control of the two-component system WalKR, which is thought to play a role in virulence and cell wall metabolism [29]. The protein is synthesized with a signal peptide (LytM_1-25_), followed by an N-terminal domain that is homologous to the staphylococcal secretory antigen A (SsaA), another WalKR controlled protein, but not to the N-terminal domain of lysostaphin. The C-terminal domain of LytM can be divided into an occluding region and a region of high similarity to the lysostaphin catalytic domain (52% amino acid identity over 106 residues). The lysostaphin active site residues are all conserved, with a central Zn^2+^ ion that is coordinated by His210, Asp214 and His293 of the catalytic domain [12]. Nevertheless, the structure strongly suggests that full length LytM cannot have significant activity, because the active site is occluded. The expected water molecule in the coordination sphere of the Zn^2+^ ion is displaced by an “asparagine switch” residue (Asn117) of the occluding region, which also blocks part of the active site cleft [12]. However, the crystal structure suggested that the catalytic domain alone should be more active than the full length protein. This was confirmed for a tryptic fragment (LytM_180-316_, previously referred to as *in vitro* activated LytM) and for the recombinantly overexpressed catalytic domain (LytM_185-316_, previously referred to as active LytM) [12,30]. In this work, we use the designation “catalytic domain” for the LytM_185-316_ fragment for consistency with the well-established lysostaphin nomenclature, even though the catalytic domain and occluding loop form the globular unit in the full length protein [12]. LytM lacks a counterpart for the cell wall targeting domain of lysostaphin (Figure 1).

The biological role of LytM is still not clear [31]. The protein was originally described as an autolysin (detected in an otherwise autolysin deficient background) [5] and reported to have glycylglycine endopeptidase activity [32]. Both we and other investigators have since then carried out experiments that cast doubt on the peptidoglycan hydrolyzing activity of full length LytM [12,31]. Nevertheless, the data clearly confirmed such activity of the catalytic fragment [12,30]. It remains to be determined whether the LytM catalytic domain can be released under physiological circumstances*.* A proteomic study of the *S. aureus* cell wall envelope fraction has identified only full length LytM (with a molecular mass of approximately 40 kDa and a pI around 6), but not in the predicted active form [33]. Although the physiological role of LytM and its catalytic domain remains uncertain, the catalytic domain has properties that could make it attractive as a potential antistaphylococcal agent. First, the protein can be easily overexpressed in *Escherichia coli* with very high yields and is easy to purify [30]. Moreover, preliminary *in vitro* experiments indicated that in certain conditions LytM_185-316_ was similarly effective as lysostaphin in clearing turbid cell wall suspensions.

Therefore, we proceeded to compare lysostaphin and LytM in a new mouse model of staphylococcal infection. The efficacy of lysostaphin was confirmed in the new model as well. Surprisingly, the catalytic domain of LytM was no more effective than control. This finding prompted us to compare properties of the two proteins in greater detail *in vitro*. Here, we report the *in vivo* observations and the *in vitro* properties of lysostaphin and LytM that might explain the different treatment outcomes.

## Results

### Chronic contact eczema model of staphylococcal infection

A new chronic dermatitis model of staphylococcal infection for *in vivo* functional studies was developed. Following standard procedures, mice were sensitized by epicutaneous application of 4-ethoxymethylene-2-phenyloxazolone (oxazolone, Sigma) on the abdomen skin. Six days later and subsequently every second day they were challenged with oxazolone applied to the ears. The treatment led to the development of chronic contact eczema in the treated ear, but not in the contralateral ear, which was left untreated as a control (Additional file 1)*.*

Preliminary experiments were run to establish a suitable *S. aureus* dose for the infection experiments. 10^6^, 10^7^, 10^8^, and 10^9^ CFUs of *S. aureus* strain LS-1 were spread on both ears of one mouse each. Mice were sacrificed two days later, ears were homogenized and *S. aureus* colony forming units (CFUs) counted. 10^6^ *S. aureus* cells per ear were sufficient to establish infection in oxazolone-treated, inflamed mouse ears, but not in non-oxazolone treated ears (data not shown).

**Figure 2:**
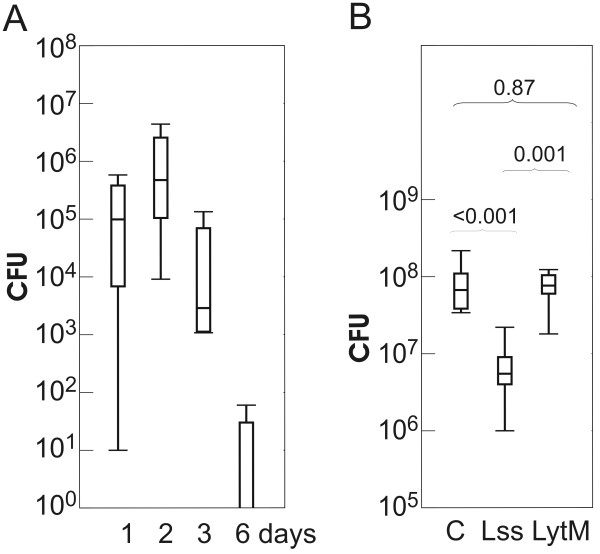
**Kinetics of*S. aureus*infection in mouse model and the effect of enzyme treatment.** Colony forming units (CFUs) after *S. aureus* infection. The data are represented in whisker-box plots. Boxes cover the second and third quartiles, and horizontal lines indicate medians. **(A)** Persistence of *S. aureus* strain LS-1 in eczematous ears of NMRI mice 1, 2, 3, and 6 days after topical application of 10^6^ *S. aureus* LS-1 per ear (n = 4/time point). **(B)** Effect of lysostaphin (Lss) and LytM_185-316_ (LytM) on *S. aureus* P1 recovery from infected mice ears as compared to the control. Twelve hours after inoculation of bacteria on ears with eczema 100 μg of lysostaphin or LytM_185-316_ (100ug each) in 50 mM glycine pH 8.0 and 10% glycerol buffer was applied to each mice ear. Ears of control mice were treated with buffer alone. Treatment was repeated 4 times every 12 hours and ears were examined 3 hours after the last treatment. The two-tailed Student's t-test (assuming equal variances in all samples) was used to calculate probabilities for the null hypothesis of equal means in pairwise comparisons. The resulting p-values are indicated above the curly brackets.

To establish the time course for the infection, 10^6^ *S. aureus* cells were applied to the oxazolone-treated, inflamed ears and to the non-oxazolone treated, contralateral control ears. At different time points following inoculation, mice were sacrificed, ears homogenized and *S. aureus* colony forming units (CFUs) counted. In non-oxazolone treated control ears, no bacteria were found after the application of 10^6^ *S. aureus* cells. In oxazolone pretreated ears, colony counts peaked two days after exposure, and bacteria were almost fully cleared six days after the inoculation (Figure 2A).

### Lysostaphin is effective in the contact eczema model, LytM_185-316_ is not

The newly developed eczema model was used for *in vivo* comparison of lysostaphin and LytM efficacies. 30 mice were divided into three groups of 10 mice each. All mice were sensitized to develop eczema, and subsequently had 10^6^ CFUs of *S. aureus* P1 cells applied to their ears to induce dermatitis. Twelve hours after inoculation of bacteria the treatment with lysostaphin and LytM_185-316_ was started. 100 μg of lysostaphin or LytM_185-316_ in 50 mM glycine pH 8.0 with 10% glycerol was applied topically to each mice ear in a volume of 20 μl. In the control group, buffer alone was used for the treatment. Ears were treated with proteins or buffer four times every 12 hours. Three hours after the last treatment mice were anesthetized, the ears dissected and the extent of infection estimated as described above. On average, the lysostaphin treatment reduced the colony count by roughly a factor of 10. In contrast to lysostaphin, LytM_185-316_ had no beneficial effect and was no better than control (Figure 2B).

We reasoned that the different treatment outcomes could reflect differences in protein stability, affinity to either peptidoglycan or other components of cell walls, or the preference for a particular pH or ionic milieu and proceeded to test the influence of all these factors *in vitro*.

### Lysostaphin is proteolytically more stable than LytM_185-316_

During treatment, lysostaphin and LytM_185-316_ were exposed both to bacterial proteases and to host proteases at the site of infection. Initial experiments demonstrated that both enzymes were stable in bacterial cultures (CFU ~10^6^). The stability to host enzymes was tested with blood and serum from rat, which were available from unrelated experiments without the sacrifice of additional animals. After 4 h incubation in 5% blood, the majority of LytM_185-316_ was degraded while the degradation of lysostaphin was minimal. Both proteins were more stable in 5% serum, but again LytM_185-316_ was less stable than lysostaphin (Additional file 2).

### Lysostaphin and LytM_185-316_ recognize different cell wall components

**Figure 3:**
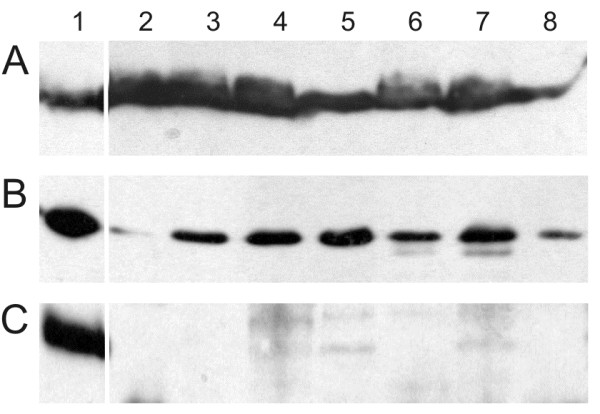
**Pulldown assay with*S. aureus*cell walls treated in various ways.** Pulldown of **(A)** lysostaphin, **(B)** LytM_185-316_ and **(C)** LytM_26-316_ with *S. aureus* cell walls treated in various ways. (1) Input, (2) sonicated crude cell walls, (3) washed crude cell walls, (4) SDS-treated cell walls, (5) TCA-treated cell walls, (6) trypsinised cell walls, (7) purified peptidoglycans (8) commercially available peptidoglycans*.* The protein that was input (lane 1) or pulled down (lanes 2–8) was visualized by Western blotting with the anti-LytM antibody.

The affinity of lysostaphin and LytM was compared in a pulldown assay using various cell wall preparations that were increasingly enriched in peptidoglycan (Figure 3). Cell walls were used either crude (lane 2) or subjected to an extra washing step (lane 3), to SDS treatment, which should remove lipid components (lane 4), to TCA treatment, which is thought to remove teichoic acids (lane 5), or to trypsin treatment, which can be expected to remove protein components from cell walls (lane 6). The pulldown assay was also carried out with “purified” peptidoglycan, which was obtained from crude cell wall preparations by a combination of the SDS-, TCA- and trypsin treatments (lane 7), and with peptidoglycan from a commercial source (Fluka) (lane 8).

In all cases, lysostaphin bound to the cell wall preparations albeit with different efficiency. Our results suggest that binding to crude cell walls was most effective, probably because of interactions between lysostaphin and non-peptidoglycan components of *S. aureus* cell walls (Figure 3A).

In contrast, LytM_185-316_ was not efficiently pulled down by crude cell wall preparations. However, when the cell walls were subjected to a washing step prior to the pulldown experiment, LytM_185-316_ could be effectively pulled down. The effect of the washing step on the cell wall preparations is not clear. It may simply reduce clumping and make cell wall structures more accessible. Alternatively it may remove a putative inhibitory factor in the unwashed cell wall sonicate. Further purification of peptidoglycan had a little effect on the outcome of the pulldown experiments. Therefore, we conclude that LytM_185-316_ binds directly to cell walls and interacts primarily with peptidoglycans, rather than with other cell wall components (Figure 3B).

Full length LytM (without predicted signal peptide, LytM_26-316_) was not efficiently pulled down by any of the peptidoglycan preparations. Traces of protein were detected in the pulldown fraction in some cases, but the effect was probably unspecific, because no systematic trend with increasing peptidoglycan purity was observed (Figure 3C).

### Lysostaphin and LytM_185-316_ bind peptidoglycan or cell walls differently

**Figure 4:**
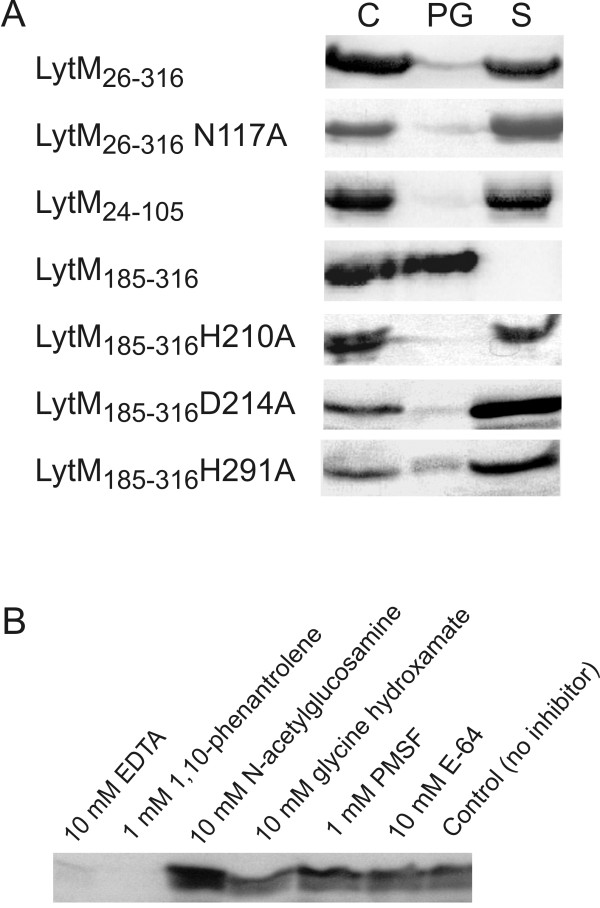
**Pulldown assay of various LytM fragments and inhibitors with purified peptidoglycans from*S. aureus*. (A)** Full length LytM and various fragments were analyzed by denaturing gel electrophoresis and Coomassie straining either directly (control, C) or after separation into peptidoglycan binding (PG) and supernatant (S) fractions. **(B)** LytM_185-316_ was incubated with peptidoglycan in the presence of various protease inhibitors and the pellet fraction after pulldown analyzed by denaturing gel electrophoresis and Western blotting.

The involvement of different regions of lysostaphin in peptidoglycan binding has been investigated earlier. The results show that lysostaphin has affinity for the pentaglycine crossbridges themselves [34], but also binds cell walls via the cell wall targeting domain [35]. In contrast, almost nothing is known about the role of different LytM fragments in peptidoglycan binding. Therefore, we investigated this question by the pulldown assay (Figure 4A). Comparing the amounts of protein in the pulldown and supernatant fractions, we found that the full length protein (LytM_26-316_) did not efficiently bind to peptidoglycan. Mutation of the Zn^2+^ ligand Asn117 to alanine, which should weaken the binding of the occluding region to the catalytic domain, did not significantly change the situation. The isolated N-terminal domain of the enzyme also failed to bind to peptidoglycan, whereas LytM_185-316_ bound efficiently. When the two Zn^2+^ ligands His210 and Asp214 were separately mutated to alanine, the binding was lost again. Changing the third Zn^2+^ ligand, His293 of the HxH motif to alanine, made the protein insoluble as reported earlier [12], so that peptidoglycan binding could not be tested. The first histidine of the HxH motif, His291, is likely to act as a general base in catalysis [11]. When this residue was mutated to alanine, peptidoglycan binding was reduced, but not fully abolished.

The requirement of an intact active site for peptidoglycan binding was also supported by inhibitor studies. We had previously shown that EDTA and 1,10-phenanthroline blocked activity, presumably by chelating Zn^2+^ ions. We now observed that both metal chelators also abolished binding of LytM_185-316_ to peptidoglycan (Figure 4B, lanes 1–2). In contrast, the weak Zn^2+^ ion chelator glycine hydroxamate and other small molecules and protease inhibitors did not interfere with peptidoglycan binding (Figure 4B, lanes 3–6). We conclude from these experiments that the accessibility and integrity of the active site is essential for the binding of the protein to peptidoglycan (Figure 4).

### Lysostaphin and LytM_185-316_ activities depend differently on pH

**Figure 5:**
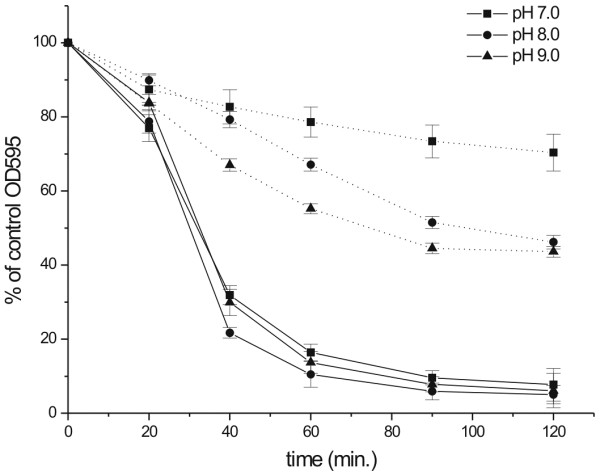
**Effect of buffer pH on lytic activity of lysostaphin and LytM**_**185-316.**_ Activity of lysostaphin (solid lines) and LytM_185-316_ (dotted lines) in 50 mM Tris buffer at pH 7.0 (squares), 8.0 (circles) and 9.0 (triangles). *S. aureus* cells were collected in the exponential growth phase, washed and resuspended in test buffer to apparent OD_595_ ~1.8. The addition of LytM_185-316_ or lysostaphin (both at 18 nM final concentration) led to cell lysis, which reduced light scattering and thus apparent OD_595_. As some decrease was also observed in the absence of enzyme, all OD_595_ values were expressed as percent of the control without enzyme.

Peptidoglycan hydrolase activities were assayed in a turbidity clearance assay, using *S. aureus* cells. Perhaps due to remaining peptidoglycan hydrolase activity in the cell wall, there was some decrease of turbidity also in control, in the absence of exogenously added enzyme. Therefore, all apparent OD values at 595 nm were expressed as percent of the control. A value close to 100% indicates a very low activity, whereas a very low OD reports highly active enzyme. Both lysostaphin and LytM_185-316_ were only marginally effective at pH 6.0 (50 mM phosphate buffer), but became much more active at pH 7.0. A further pH increase to the range between 7.0 and 9.0 (50 mM Tris–HCl) had little effect on the activity of lysostaphin, but enhanced the activity of LytM_185-316_. Even at pH 9.0, incubation with LytM_185-316_ lysed fewer cells than incubation with the equivalent amount of lysostaphin, particularly at late time points, possibly because of the lower stability of LytM_185-316_ (Figure 5).

### Lysostaphin and LytM_185-316_ activities depend very differently on ionic strength

Investigating the pH dependence, we noticed a dramatic dependence of the lysis efficiency on the buffer. For example, the activity of LytM_185-316_ was much higher in 20 mM than in 50 mM Tris–HCl (both pH 8.0), and increased further when Tris was replaced with glycine at pH 8.0. However, glycine did not seem to act as an allosteric activator, because it did not enhance the activity when it was added in the presence of other buffer substances. Similar observations were made with other buffer components (Additional file 3).

**Figure 6:**
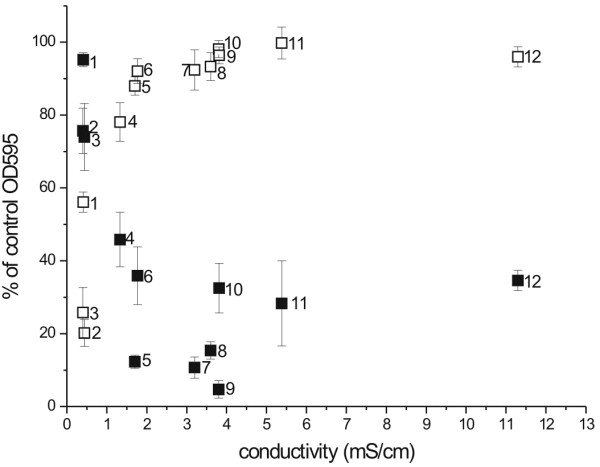
**Effect of various buffers on lytic activity of lysostaphin and LytM**_**185-316**_. Lysis by lysostaphin (closed squares) and LytM_185-316_ (open squares) was done in following buffers: (1) dd water, (2) glycine-NaOH, (3) D,L-alanine-NaOH, (4) diglycine-NaOH, (5) bicine-NaOH, (6) triglycine-NaOH, (7) Tris-HCl, (8) hepes-NaOH, (9) phosphate buffer, (10) L-arginine-HCl, (11) L-glutamic acid-NaOH, (12) diaminopimelic acid-NaOH. All buffers were 50 mM with pH adjusted to 8.0 and data were collected after 60 min of reaction.

A clear pattern emerged only when lysis activities of LytM_185-316_ and lysostaphin were correlated with the conductivity of the buffers (Figure 6). Lysostaphin degrades *S. aureus* cell walls inefficiently in low conductivity buffers, but becomes more efficient in buffers of higher conductivity. In contrast, LytM_185-316_ works best at low conductivity, and is almost ineffective in high conductivity buffers. The transition region for both effects is around 2 mS/cm, which corresponds roughly to a total ion concentration of 15–20 mM for singly charged cations and anions and typical mobilities (Figure 6).

**Figure 7:**
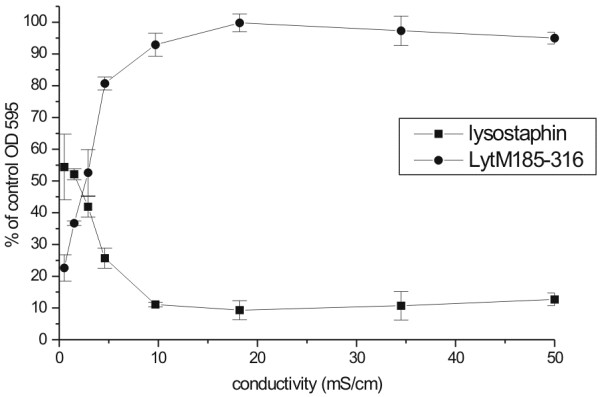
**The effect of ionic strength of reaction buffer on lytic activity of lysostaphin and LytM**_**185-316**_. Lysis was done in standard conditions (see Material and Methods) in 20 mM glycine buffer pH 8.0 supplemented with 0 to 500 mM NaCl. Conductivity of the reaction was measured at room temperature after addition of *S. aureus* cells. Presented results were collected after 60 min of lysis reaction at 37°C.

Conductivity reflects both ion concentration and mobility. We reasoned that ionic strength was more likely than conductivity to influence protein activity, and therefore varied conductivity systematically by changing the concentration of sodium chloride between 0 and 500 mM. Lysostaphin and LytM_185-316_ activities were again dependent on the ionic strength in the expected manner, but conductivity was more directly correlated with ionic strength in this experiment (Figure 7).

The influence of ionic strength could also be demonstrated in a different way that was more directly related to the *in vivo* experiments. The low lytic efficiency of lysostaphin in glycine buffer could be overcome by addition of 25 to 100% of serum. Conversely, the addition of 25% or more serum to optimal reaction conditions for LytM_185-316_ (50 mM glycine-NaOH) completely abolished the activity of enzyme (data not shown).

The analysis of MIC and MBC for lysostaphin and LytM_185-316_ confirmed the above conclusions. The MIC for lysostaphin was around 0.0015-0.003 μg/ml, but inhibition of bacterial growth was not observed even with 5 μg/ml of LytM_185-316_. The MBC of lysostaphin was approximately 0.15 μg/ml in CASO broth and glycine buffer in agreement with previous data [36]. LytM_185-316_ had an MBC around 0.3 μg/ml in the low ionic strength glycine buffer, but did not exhibit bactericidal activity in CAMH or CASO broth growth media which have conductivity 18 mS/cm.

## Discussion

Lysostaphin treatment of *S. aureus* infection has been reported earlier. In a cotton rat model, *S. aureus* nasal colonization has been eradicated by this enzyme [26]. In the mouse, *S. aureus* systemic infections have been successfully treated [25] and biofilms have been effectively eliminated from a catheterized jugular vein [24]. The chronic dermatitis model of staphylococcal infection reported in this paper differs significantly from the earlier models and therefore represents an independent confirmation for the efficacy of lysostaphin. The lack of efficacy of the LytM_185-316_ treatment was initially surprising in light of previously observed comparable activity of lysostaphin and LytM_185-316,_ though in experiments carried out in low salt buffers. As a result of this work, we now know that LytM_185-316_ differs from lysostaphin in several ways that could all explain the outcome of the mouse experiments.

### Stability

LytM_185-316_ is a very stable protein in extracts of *Escherichia coli*, where the protein can be effectively produced recombinantly. The protein is also stable against staphylococcal proteases, just like lysostaphin. However, there are stability differences in serum and blood. This would obviously be relevant if lysostaphin or LytM were used systemically. As we are not sure to what extent the proteolytic stabilities in blood or serum reflect the situation in tissues with eczema, the influence of this factor on the overall treatment income is not clear though should not be neglected.

### Binding

Both lysostaphin and LytM_185-316_ bind the pentaglycine crossbridges of *S. aureus* peptidoglycan. Both proteins recognize the crossbridges themselves, probably at least in part by interactions with the active site cleft. Lysostaphin has an extra cell wall targeting (CWT) domain which provides affinity. There is no counterpart in LytM (or LytM_185-316_), and therefore we originally expected that the N-terminal domain of the full length protein might play a similar role, especially in the light of the homology to SsaA. However, our experiments argue against this possibility, because full length LytM does not bind peptidoglycan.

### Modular structure

LytM_185-316_ binds purified peptidoglycan the most effectively. The opposite is true for lysostaphin, which seems to recognize other cell wall components as well. It has previously been reported that deletion of the CWT domain in lysostaphin does not interfere with the endopeptidase activity of the enzyme, but abolishes its ability to distinguish between *S. aureus* and *S. staphylolyticus*[37]. As the peptidoglycans of the two bacterial species are identical [38], it suggests the recognition of non-cell wall components by CWT. Irrespective of which part of the lysostaphin protein provides the affinity to non-peptidoglycan cell walls, the ability of the protein to bind to crude cell walls is clearly helpful to lyse intact cells and seems to provide lysostaphin with an advantage as a protein drug. LytM is an autolysin, which is produced by the cell and delivered to the cell wall from “inside” while lysostaphin is a bacteriocin that approach target cells from the “outside”. In the treatment model, the approach of the peptidoglycan hydrolases to cell walls is necessarily from the outside, again favouring lysostaphin over any LytM fragment.

### Ionic milieu

Perhaps the most crucial factor to explain the different treatment outcomes is the very different response of the two proteins to the ionic milieu. We do not know the precise ionic milieu of the contact eczema model of *S. aureus* infection, but suspect that it belongs to the high ionic strength regime, which would certainly apply for serum. If this is true, the ionic milieu in the mouse eczema could explain differences in treatment outcomes between lysostaphin preferring higher concentrations of salts for its activity and LytM being strongly inhibited in such environment.

## Conclusions

Perhaps the strongest predictor of the mouse experiments is the biology of lysostaphin and LytM. As a bacteriocin, lysostaphin is evolved for the lysis of *S. aureus* cell walls. In contrast, LytM as an autolysin should be evolved to have its activity under tight control. We expected this to apply for the full length enzyme, but hoped to bypass this step by the artificial activation that removes the N-terminal domain and the occluding region. Apparently, this does not suffice, because there are differences at several other levels which reflect the different *in vivo* roles of lysostaphin and LytM. We conclude that the use of LytM_185-316_ as an antibacterial agent is a more remote possibility than originally envisaged and that efforts to develop antibacterial peptidoglycan hydrolases should perhaps be concentrated on proteins that act as bacteriocins rather than autolysins.

## Methods

### Bacterial cultures

Bacteria were grown in CASO broth (Fluka) at 37°C with strong aeration from a 100-fold dilution of overnight cultures. Three strains of *S. aureus* were used in the studies. The LS-1 is an arthritogenic strain originally isolated from swollen mouse joint [39]. The 8325–4 strain is a derivative of NCTC 8325, which has been cured of resident prophages and has low production of coagulase and surface adhesions [40]. The P1 strain was isolated from a rabbit inoculated with ATCC 25923 and has better adherence than 8325–4 to endothelial cells (a generous gift from prof. T.J. Foster, Trinity College, Dublin, Ireland) [41]. The LS-1 strain was used to develop the eczema model, while the P1 strain was used for a comparison of enzyme efficacies in the eczema model. Strain 8325–4 was used for all *in vitro* assays (pulldown, lysis, stability). The susceptibilities of the 8325–4 and P1 strains towards LytM and lysostaphin were comparable.

### Proteins

A fragment of DNA corresponding to the LytM_24-105_ protein was amplified by PCR from the previously described full length LytM clone [12] inserted into the pET15mod vector and called pET15modLytM_24-105_. The construct coded for the LytM fragment fused to an amino-terminal histidine tag and could be expressed in soluble form in *E. coli* strain BL21(DE3). Protein expression was induced during the logarithmic growth phase of the bacteria (OD_595_ of 0.8) by the addition of 1 mM IPTG and continued for 4 h at 25°C. The recombinant protein was purified by affinity chromatography on a Ni^2+^ loaded, nitrilo-triacetic acid (NTA) agarose column (Qiagen), followed by gel filtration on a Sephacryl S-200 column (Amersham Bioscience). LytM_26-316_, LytM_99-316_, LytM_185-316_ and all point mutants were expressed and purified as previously described [12]. Lysostaphin (mature form) was purchased from Sigma and used without further purification.

### Antibodies

Polyclonal antibodies against LytM_185-316_ were raised in rabbit (Pineda Antibody Service, Berlin, Germany). Antibody purification was performed by affinity to LytM_185-316_ protein coupled to CNBr-activated Sepharose 4B (Amersham Bioscience) according to the manufacturer’s instructions. After washing, antibodies were eluted with 100 mM glycine pH 2.7. The pH of the eluent was immediately neutralized by the addition of 1/10 volume of 2 M Tris–HCl pH 8.0. The concentration of the antibodies in the eluent was estimated based on the absorption at OD_280_.

### Western blot hybridization

Proteins separated by SDS-PAGE were transferred onto ECL membrane (Amersham Bioscience) by semidry transfer and then incubated with 0.5 μg/ml purified antibodies against LytM_185-316_ protein. Goat anti-rabbit peroxidase-conjugated secondary antibodies (Sigma) were detected using Western Blot Luminol Reagent (Santa Cruz Biotechnology).

### LytM stability

Supernatants from 1 ml cultures of *S. aureus* at late exponential phase were concentrated, mixed with 2 μg of LytM_26-316_, and incubated overnight at 37°C. Proteins were separated on SDS-PAGE and used for Western blot hybridization. To assess the stability of lysostaphin and LytM_185-316_ in buffer with addition of blood or serum (from rat) enzyme was mixed with 5% or 50% blood or serum in 50 mM glycine pH 8.0, and incubated at 37°C. Protein samples were collected after 1 and 4 h, separated by SDS-PAGE and used for Western blot hybridization.

### Cell wall treatment

Late exponential phase cultures of *S. aureus* grown in CASO Broth medium were harvested by centrifugation, resuspended in buffer A (20 mM Tris–HCl pH 7.5) and autoclaved for 20 min. Crude extract was obtained after sonicating the cells for 3 min. The accessory wall polymers were removed by the following methods. SDS treated walls were boiled in 4% SDS for 30 min. Trypsinized walls were prepared by 8 h trypsin digest (0.5 mg/ml) at 37°C. Trichloroacetic acid (TCA) treatment was done by 48 h incubation in 10% TCA at 4°C. After each of these treatments, cell walls were extensively washed in buffer A. Purified peptidoglycans were prepared as described previously [12] by combining all methods described above. Alternatively, *S. aureus* peptigdoglycan was purchased from Fluka Biochemika.

### Pulldown peptidoglycan binding assay

To assess binding, 2 μg of protein was mixed with cell walls or peptidoglycans (100 μg) and incubated at room temperature for 15 min. Then, soluble and insoluble fractions were separated by centrifugation and peptidoglycans were washed with 1 ml of buffer A. Soluble fractions and washed peptidoglycans were mixed with loading buffer separated by SDS-PAGE and analyzed by Western blot hybridization. Final concentrations of 10 mM EDTA, 1 mM 1,10-phenanthroline, 10 mM N-acetylglucosamine, 10 mM glycine hydroxamate, 1 mM PMSF and 1 mM E-64 were used to test the influence of these compounds on peptidoglycan binding.

### Cell lysis assay

*S. aureus* cells collected at the exponential growth phase were washed and suspended in buffer A supplemented with 200 μg/ml erythromycin. Then the cells were diluted to an apparent OD_595_ of 1.8 with an appropriate buffer. Enzymes were added to the final concentration of 18nM and 200 μl of reaction transferred onto the microtiter plate. Plates were incubated at 37°C with 2 s shaking every 5 min. OD of the suspension was checked at the wavelength of 595 nm at 0, 20, 40, 60, 90 or 120 min. after starting the reaction. Ionic strength of the reaction milieu (cells resuspended in appropriate buffer) was measured using conductivity meter MeteLab CDM230 (Radiometer Analytical, France) at the beginning of the tests. Lytic activity was calculated as a per cent of control OD_595_ (the same samples as for reaction but without enzymes). Each experiment was repeated twice in quadruplicate.

### Determination of minimal inhibitory concentration (MIC) and minimal bactericidal concentration (MBC)

Both parameters were determined generally as described by Kusuma and Kokai-Kun [36]. For MIC determination by the microdilution method, 100 μl of Cation-Adjusted Mueller-Hinton broth were inoculated with ~10^4^ *S. aureus* cells (strain 8325–4) and enzyme concentrations between 4 and 0.0015 μg/ml were tested. For MBC determination, ~10^6^ CFU/ml of *S. aureus* cells (strain 8325–4) in either CASO broth or in 50 mM glycine pH 7.5 were incubated with between 10 to 0.15 μg/ml of enzyme. For lysostaphin, but not for LytM, the buffer was supplemented with 150 mM NaCl to make digestion conditions optimal for the enzyme.

### Animal experiments

Ethical permission for animal experiments was obtained from the Animal Research Ethics Committee of Göteborg University. Throughout the experiments the animals were under control of the veterinarian. No differences in animal behavior and general state of health were observed between the control and experimental groups.

### Induction of chronic contact eczema in mice

NMRI mice were sensitized by epicutaneous application of 150 μl of a mixture of ethanol and acetone (3:1) containing 3% of 4-ethoxymethylene-2-phenyloxazolone (oxazolone, Sigma) on the abdomen skin. Six days later, and subsequently every second day, all the mice were challenged on both sides of one ear with 30 μl 1% oxazolone dissolved in olive oil. The mice received altogether 4 oxazolone challenges on the ear. This procedure leads to chronic, eczematous skin inflammation characterized macroscopically by swelling, redness and superficial desquamation and microscopically by influx of inflammatory cells (Additional file 4).

### Infection kinetics

The day following the last application of oxazolone, the mice were briefly anaesthetized, and *S. aureus* in a volume of 10 μl was spread on the skin surface of the inflamed ear. In the first experiment four mice with dermatitis were subjected to skin infection in one ear while the contra lateral ear was used as a control. *S. aureus* strain LS-1 at 10^6^, 10^7^, 10^8^, and 10^9^ CFU (colony forming unit) was spread on each ear, and the mice were sacrificed two days later. In the second experiment, the kinetic of infection was assessed. Twenty mice with dermatitis on one ear were exposed to 10^6^ CFU *S. aureus* strain LS-1*.* Groups of five mice each were sacrificed at 1, 2, 3, and 6 days following exposure to bacteria. One mouse ear/group was subjected to histological examination (Additional file 4) and the rest 4 ears/group were subjected to enumeration of staphylococci.

### Comparison of lysostaphin and LytM_185-316_ in the mouse model

In the last *in vivo* experiment the staphylococcal strain P1 (10^6^/ear) was used to infect ears of mice with eczema. Twelve hours after inoculation of bacteria the treatment with proteins was started; 100 μg of lysostaphin or LytM_185-316_ in 50 mM glycine pH 8.0 and 10% glycerol buffer was applied to each mouse ear in a volume of 20 μl. In the case of control mice buffer alone was used for the treatment. Ears were treated with proteins or buffer four times every 12 hours. Three hours after the last treatment mice were anesthetized and the ears dissected. The ears were washed with alcohol to remove surface bound bacteria, kept on ice, homogenized and diluted in PBS. One hundred microliter of the homogenate from various dilutions was then transferred to agar plates, containing 7.5% sodium chloride. After incubation at 37°C for 24 hours the colony forming units were counted. 10 mice were used in the control group and in each treatment group.

Prior to the *in vivo* use, staphylococci were cultured for 24 hours on blood agar plates, re-inoculated and grown on fresh blood agar plates for another 24 hours, harvested, and stored frozen at −20°C after suspending aliquots in phosphate-buffered saline (PBS) supplemented with 5% bovine serum albumin and 10% dimethyl sulphoxide. Before application on ears, staphylococcal suspensions were thawed, bacteria washed in PBS and diluted in PBS to achieve the appropriate concentration of the staphylococci. To determine the CFU, aliquots of staphylococcal suspensions were subjected to dilution, plating on blood agar and enumeration.

## Authors’ contributions

IS carried out the molecular and biochemical studies, participated in the animal experiment and drafted the manuscript. I-MJ carried out the animal experiments. AT, MB participated in the design and coordination of experiments and contributed to drafting the manuscript. IS, I-MJ and MB read and approved the final version of manuscript, AT read and approved an earlier version prior to his untimely death.

## Supplementary Material

Additional file 1 Picture of mouse ears untreated (on the left) and treated (on the right) with oxazolone. Click here for file

Additional file 2 Stability of LytM_185-316_ and lysostaphin. Proteins were incubated without (1) or with concentrated, conditioned *S. aureus* media (2), 5% (4) or 50% (5), blood and 5% (6) or 50% (7) serum. After incubation proteins were separated by SDS-PAGE electrophoresis and detected by Western blot hybridization with anti-LytM antibodies. Click here for file

Additional file 3 Time course of *S. aureus* 8325–4 cell lysis by LytM_185-316_ and lysostaphin in various conditions. (A) Influence of glycine. Lysis experiments were done in 100 mM glycine-NaOH, pH 8.0, 50 mM Tris-HCl, pH 8.0 and 100 mM glycine in 50 mM Tris-HCl pH 8.0. (B) Influence of mono-, di- and triglycine. Buffers were made as 50 mM with pH adjusted to 8.0 with NaOH. For comparison lysis in dd water was also checked. (C) Influence of various aminoacids. 50 mM L-arginine-HCl, D,L-alanine-NaOH, L-arginine-HCl, L-glutamic acid-NaOH, diaminopimelic acid (DAP)-NaOH of pH 8.0 were tested. Lysis experiments were performed as described in Material and Methods. Click here for file

Additional file 4 Histological examination of mouse ear during the development of eczema and *S. aureus* infection. (A) section of control ear, (B) section 2 days after *S. aureus* infection; massive invasion of inflammatory cells can be observed (indicated with open arrows). Click here for file
