# Supplementary figures and images for: Anti-staphylococcal activities of lysostaphin and LytM catalytic domain
Source: BMC Microbiol. 2012 Jun 6;12:97. doi: 10.1186/1471-2180-12-97 (PMC3413552; doi:10.1186/1471-2180-12-97)

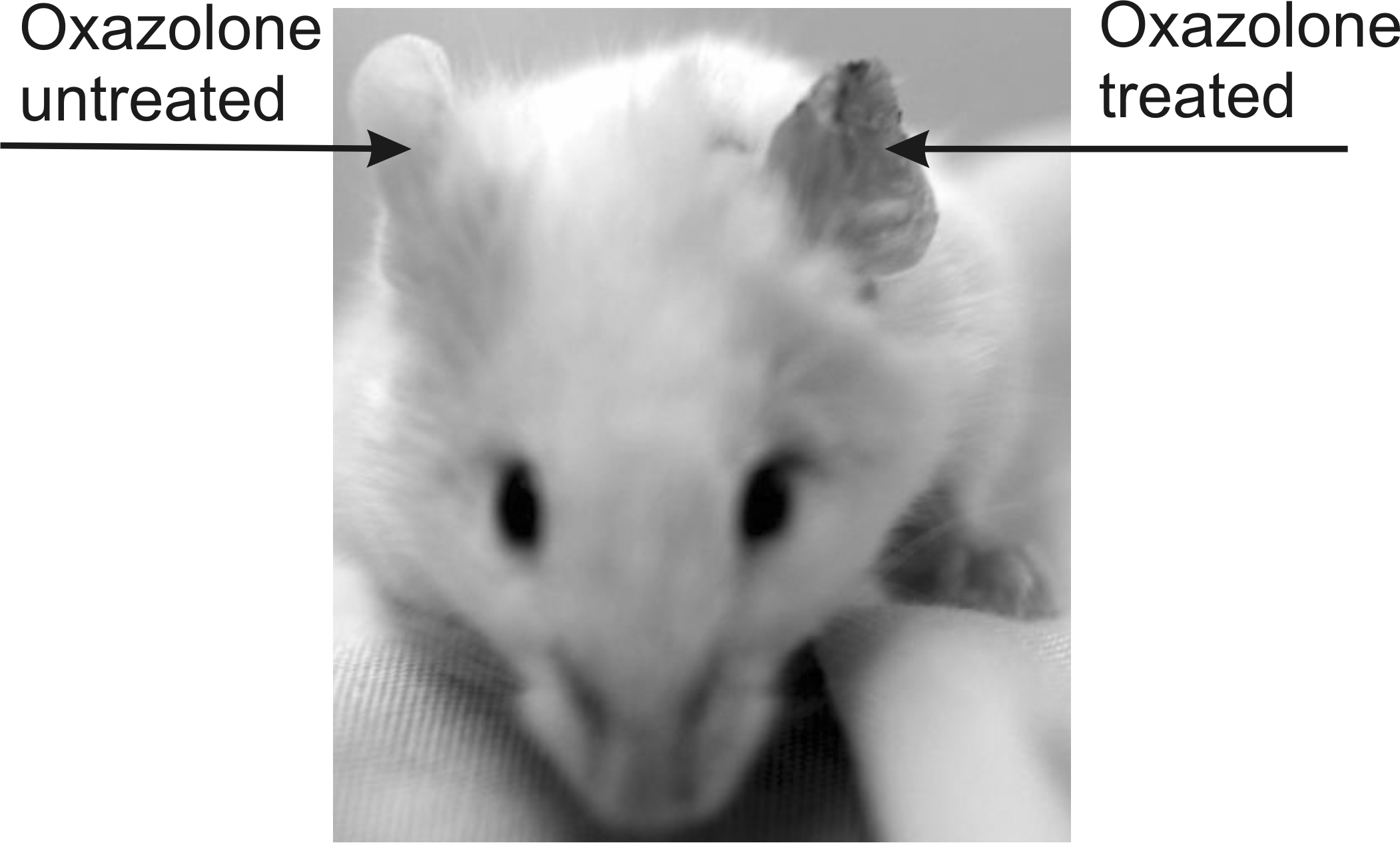

Supplement: Additional file 1 — Picture of mouse ears untreated (on the left) and treated (on the right) with oxazolone. [file 1471-2180-12-97-S1.tiff]

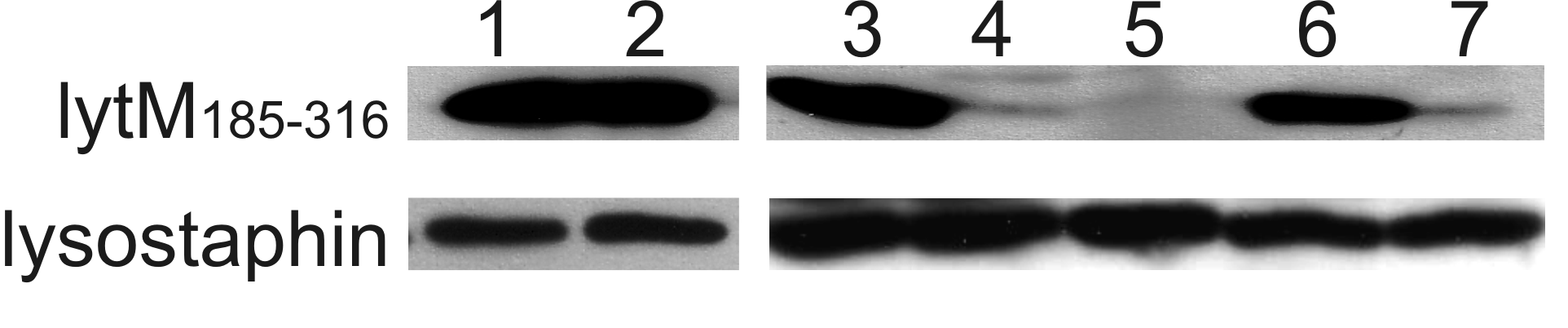

Supplement: Additional file 2 — Stability of LytM185-316 and lysostaphin. Proteins were incubated without (1) or with concentrated, conditioned S. aureus media (2), 5% (4) or 50% (5), blood and 5% (6) or 50% (7) serum. After incubation proteins were separated by SDS-PAGE electrophoresis and detected by Western blot hybridization with anti-LytM antibodies. [file 1471-2180-12-97-S2.tiff]

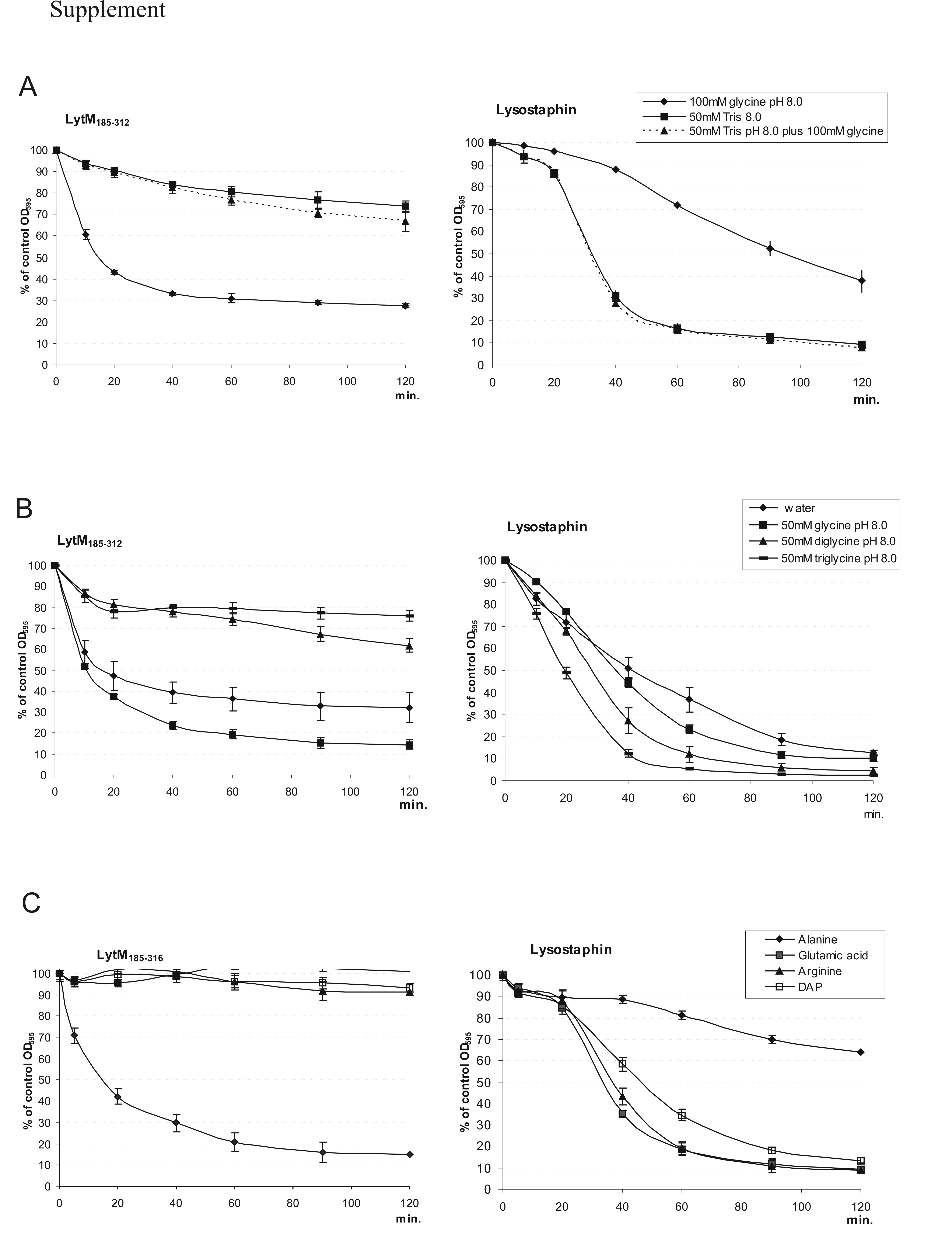

Supplement: Additional file 3 — Time course of S. aureus 8325–4 cell lysis by LytM185-316 and lysostaphin in various conditions. (A) Influence of glycine. Lysis experiments were done in 100 mM glycine-NaOH, pH 8.0, 50 mM Tris-HCl, pH 8.0 and 100 mM glycine in 50 mM Tris-HCl pH 8.0. (B) Influence of mono-, di- and triglycine. Buffers were made as 50 mM with pH adjusted to 8.0 with NaOH. For comparison lysis in dd water was also checked. (C) Influence of various aminoacids. 50 mM L-arginine-HCl, D,L-alanine-NaOH, L-arginine-HCl, L-glutamic acid-NaOH, diaminopimelic acid (DAP)-NaOH of pH 8.0 were tested. Lysis experiments were performed as described in Material and Methods. [file 1471-2180-12-97-S3.tiff]

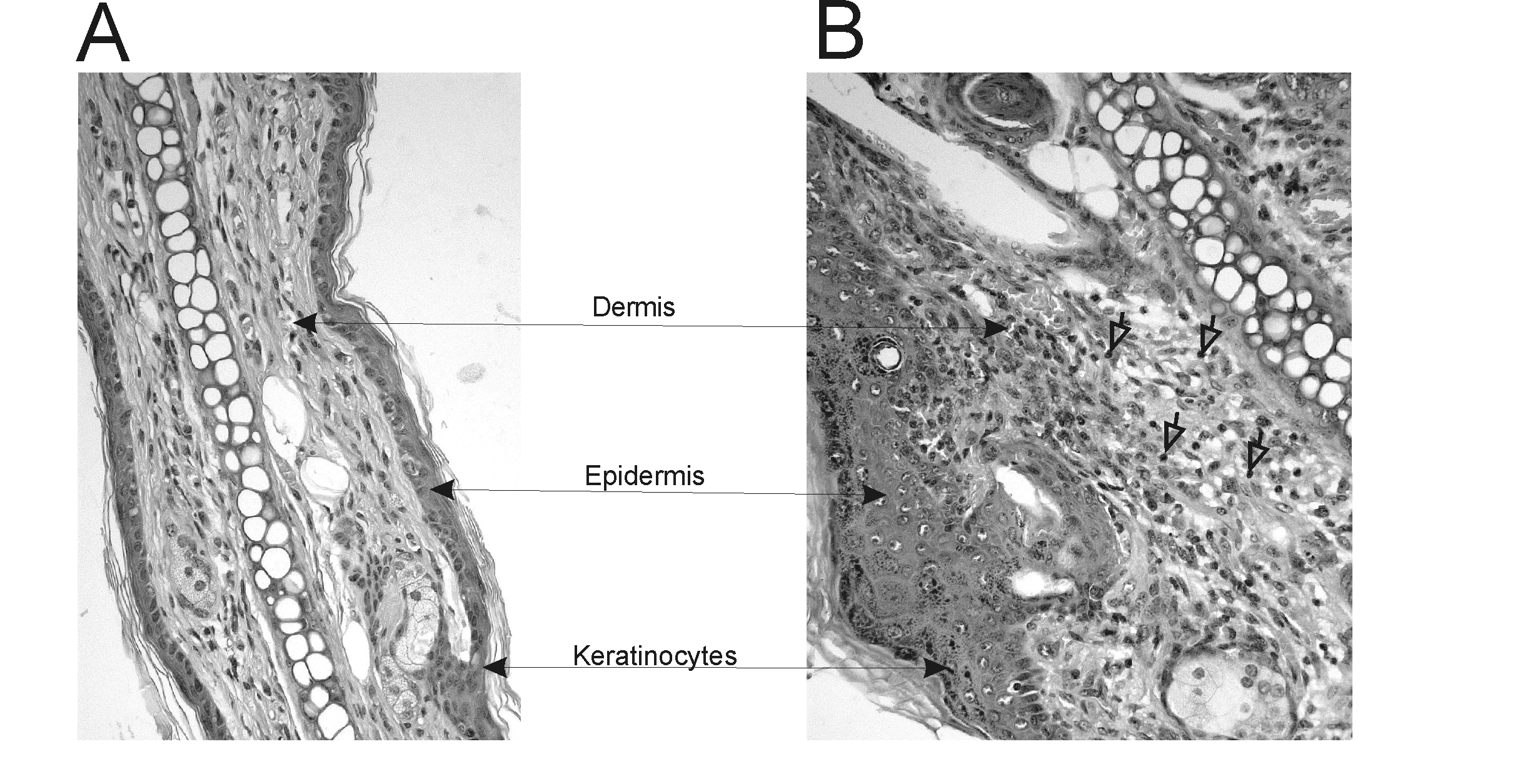

Supplement: Additional file 4 — Histological examination of mouse ear during the development of eczema and S. aureus infection. (A) section of control ear, (B) section 2 days after S. aureus infection; massive invasion of inflammatory cells can be observed (indicated with open arrows). [file 1471-2180-12-97-S4.tiff]
